# Medical student recognition of benign anorectal conditions: the effect of attending the outpatient colorectal clinic

**DOI:** 10.1186/1471-2482-14-95

**Published:** 2014-11-19

**Authors:** Constantine P Spanos, Apostolos Tsapas, Manolis Abatzis-Papadopoulos, Eleni Theodorakou, Giorgios N Marakis

**Affiliations:** 1st Department of Surgery, Aristotelian University School of Medicine, 15 Fitziou Street, N751, Panorama-Thessaloniki, 55236 Greece

## Abstract

**Background:**

Benign anorectal conditions are fairly common. Physicians of various specialties usually see patients with these conditions before being referred to colorectal specialists, frequently with an incorrect diagnosis.

We sought to evaluate the effect of attending an outpatient colorectal clinic by medical students on the diagnostic accuracy of these conditions.

**Methods:**

Over a 1-year period, medical students were randomized into a group that attended the clinic, and one that did not. Both groups were shown images of six common benign anorectal conditions. The overall diagnostic accuracy as well as the diagnostic accuracy for each one of these conditions was prospectively evaluated for both groups.

**Results:**

Nineteen students attended clinic and 17 did not. Overall diagnostic accuracy was 80.6% for students attending clinic and 43.1% for non-attending students. (p < 0.05) In the attending group, diagnostic accuracy was significantly greater for prolapsed internal hemorrhoids (73.6% *versus* 35.2%, p < 0.05), thrombosed external hemorrhoid, (73.6% *versus* 17.6%, p < 0.05) fissure (100% *versus* 47%, p < 0.05), and anal tags (68.4% *versus* 11.7%, p < 0.05%).

**Conclusion:**

Exposure to these conditions during surgical clerkships in medical school may help future specialists provide better care for patients with benign anorectal disorders.

## Background

Benign anorectal disorders such as hemorrhoids, fissure, abscesses, fistulae and condyloma are fairly common; general practitioners, gastroenterologists and internists will initially see a great proportion of these conditions. Most, if not all, of these disorders are treated by general or colorectal surgeons.

Most patients seeking consultation with colorectal surgeons are referred (mostly by the above-mentioned specialists) as having “hemorrhoids”. However, most are found to have other benign anal pathology. It is reasonable to state that misdiagnosis of benign anal disorders leads to delay in the definitive treatment of these disorders, which may be preceded by a slew of unnecessary referrals, tests and procedures. Increased healthcare costs may result [1].

It is imperative that non-colorectal physicians make accurate diagnoses of benign anorectal disorders and provide basic treatment, as there are a limited number of colorectal specialists; exclusive care of these patients by such a specialist would possibly be impractical and unnecessary [2]. In addition, these physicians should be able to refer patients for specialized consultation to a colorectal surgeon, when necessary.

Even though graduating general surgeons are expected to be proficient in the diagnosis of common anorectal pathology, surgical training programs may not provide trainees with adequate exposure to anorectal disorders. This may be secondary to small numbers of anorectal cases performed by surgical residents in combination with a rather limited exposure to the outpatient colorectal clinic [2].

Several reports have demonstrated significant misdiagnosis rates for benign anorectal disorders by general surgeons and general practitioners [3].

In addition, medical school curricula may provide even less exposure to anorectal anatomy, as well as pathology. One would hypothesize that therein lays the root of the problem.

The goal of our study was to prospectively evaluate the effect of attendance of an outpatient colorectal clinic by medical students on the diagnostic accuracy of common benign anorectal disorders.

### Ethics

The institutional review board/ethics committee of the Papageorgiou Hospital approved the study. All patients signed a written informed consent. Data collection and analysis was performed in compliance with the Helsinki Declaration. Written consent for usage of photographs for the study was obtained from the patients.

## Methods

In our department, a dedicated lecture on benign anorectal disorders is held for 4^th^ year medical students on their surgical rotation. A similar lecture is given in the 6^th^ (final) year. During these lectures, images of benign anorectal lesions are typically shown. During the present study, medical students in their final year were accrued over a 1-year period. These students were rotating through their surgical clerkship during the time of the study. There were three, 12-week surgical clerkships/semesters during the year. At the beginning of each clerkship, students were randomized into two groups. The first group attended the outpatient colorectal clinic while the other did not. Each student ended up attending 3 clinic sessions. All patient encounters in the attending group were conducted under direct supervision of a colorectal specialist certified by the American Board of Colon & Rectal Surgeons (author CPS). The specialist provided didactics regarding history taking, physical examination, diagnosis and treatment during each clinic session. The specialist made the final diagnosis in clinic. At the end of each semester, images of 5 common benign anal disorders were shown to both groups. These images included prolapsed internal hemorrhoids, thrombosed external hemorrhoid, full-thickness rectal prolapse, anal fistula, and anal fissure. In addition an image of anal tags was shown. The images were shown to three board-certified colorectal specialists and were validated when all images were correctly identified.

**Figure 1:**
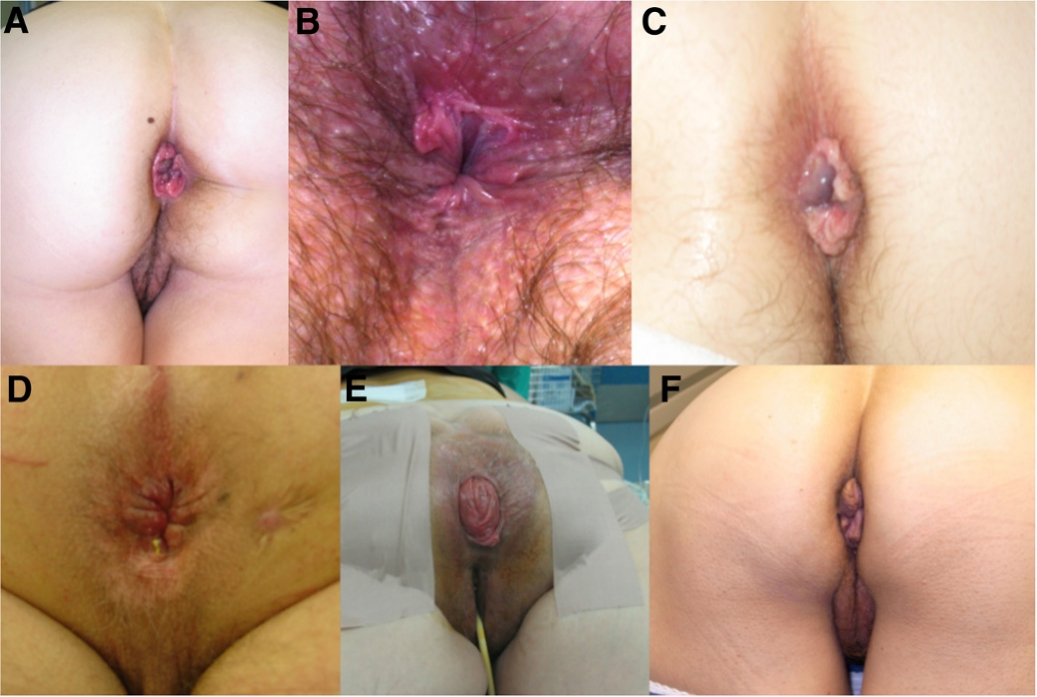
**Pictures of benign anorectal conditions shown to medical students. A**: prolapsed internal hemorrhoids, **B**: anal fissure, **C**: thrombosed external hemorrhoid, **D**: anal fistula, **E**: full-thickness rectal prolapse, **F**: anal tags.

Slides of the images were shown simultaneously to both groups of students for 1 minute (Figure 1). All students were then asked to provide a written diagnosis on an answer sheet. Each answer sheet had a blank line, where the written diagnosis was provided. At the end of the image display, all answer sheets were collected in a sealed envelope and were scored by an independent auditor. At the conclusion of the study, three such “tests” had been administered; one per semester. We calculated an overall diagnostic score based on the number of correct answers provided for the six pictures demonstrated to each student. We also calculated the number of students in each group that answered correctly more than half of the questions (≥4).

We used descriptive statistics to summarize the diagnostic accuracy and compare the differences between the two groups. We utilized independent samples t-test and Chi-square test to test for differences between the two groups. Statistical significance level was set at 0.05. All analyses were carried out using STATA version 12.1 (Stata Corporation, College Station, TX).

## Results

Thirty-six students were accrued. There were 19 students in the “Attending” (Group A) and 17 students in the “Non-attending” (Group B). This group served as the control group. All students in Group A observed prolapsed internal hemorrhoids, anal fissure and anal tags in clinic. Anal fistula was observed by 89.4%, external thrombosed hemorrhoids by 78.9%, full-thickness rectal prolapse by 31.5%, condyloma by 26.3%, and peri-anal abscess by 26.3% (Table 1).

**Table 1:** **Benign anorectal disorders observed by students attending the outpatient colorectal clinic**

Disorders seen	Attending students (Group A)
Prolapsed Internal Hemorrhoids	19/19 (100%)
Thrombosed External Hemorrhoid	15/19 (78.9%)
Fistula	17/19 (89.4%)
Fissure	19/19 (100%)
Prolapse	6/19 (31.5%)
Anal Tags	19/19 (100%)
Condyloma	5/19 (26.3%)
Abscess	5/19 (26.3%)

Overall diagnostic accuracy in Group A was significantly higher than in Group B (80.6% *versus* 43.1%, p < 0.05). Regarding individual anorectal disorders, students in Group A demonstrated significantly better diagnostic accuracy with internal prolapsed hemorrhoids (73.6% *versus* 35.2%, p < 0.05), external thrombosed hemorrhoids (73.6% *versus* 17.6%, p < 0.05), anal fissure (100% *versus* 47%, p < 0.05) and anal tags (68.4% *versus* 11.7%, p < 0.05%). Diagnostic accuracy was not significantly different between Group A and B regarding rectal prolapse (89.4% *versus* 88.2%, p = NS) and anal fistula (78.9% *versus* 58.8%, p = NS). Table 2 summarizes accuracy of each anorectal condition between the two student groups.

**Table 2:** **Diagnostic accuracy of medical students attending clinic compared with students not attending for each condition**

Diagnostic accuracy	Attending students	Non-attending students	
**Prolapsed Internal Hemorrhoids**	14/19 (73.6%)	6/17 (35.2%)	p < 0.05
**Thrombosed External Hemorrhoid**	14/19 (73.6%)	3/17 (17.6%)	p < 0.05
**Rectal Prolapse**	17/19 (89.4%)	15/17 (88.2%)	p = NS
**Fissure**	19/19 (100%)	8/17 (47%)	p < 0.05
**Fistula**	15/19 (78.9%)	10/17 (58.8%)	p = NS
**Anal Tags**	13/19 (68.4%)	2/17 (11.7%)	p < 0.05
Total Accuracy	80.6%	43.1%	p < 0.05

NS = Not significant.

Four out of 19 (21%) of students in Group A were able to correctly identify all anorectal conditions. Two out of 17 (11.7%) of students in the Group B misidentified all anorectal conditions. The minimum number of anorectal conditions identified by students in Group A was 3 out of 6 (5.2% of students in Group A).

The overall diagnostic accuracy score was 2.6 ± 0.3 for the control group and 4.8 ± 2.2 for the intervention group (p < 0.001). Seventeen students (89%) in Group A identified correctly at least four (≥4) of the pictures demonstrated, compared to only two students in the Group B (12%) (p < 0.001).

## Discussion

Benign anorectal conditions are fairly common; the estimated prevalence of symptomatic hemorrhoids alone in Western countries is 4.4-5% [4]. A fair number of patients with anorectal conditions are initially seen by non-colorectal specialists. These include general practitioners, internists, emergency physicians, gastroenterologists and gynecologists. Misdiagnosis of these conditions is common. Grucela et al. demonstrated that overall diagnostic accuracy of benign anorectal disorders was 70.4% for surgeons, and less than 50% for other specialties [1]. Significant rates of misdiagnosis of benign anorectal conditions have also been noted among surgical trainees. Miller et al. demonstrated that surgical trainees, failed to diagnose anal fissures 38% of the time [2].

Several hypotheses for the high rates of misdiagnosis can be made. For surgical trainees, it may be the relative lack of exposure and education regarding benign anorectal disorders. The average surgical trainee in the United States performs approximately 30 anorectal cases throughout his or her 5-year training [5, 6]. Lack of attendance of the outpatient colorectal clinic may also be contributory [2]. Furthermore, exposure, education and instruction regarding benign anorectal disease in medical school curricula are variable at best; most students acquire knowledge of these disorders during lectures and through textbooks. In addition, basic tenets of the anorectal exam such as the digital rectal exam (DRE) are performed by medical students less frequently than before. It has been reported that the number of DREs performed by medical students in the UK dropped between 1990 and 2000 from a median of 30 ± 11 to 5 ± 3 [7]. In a systematic assessment of the utilization and utility of DRE across medical students and a spectrum of specialists in clinical practice, DRE was underutilized [8]. Approximately 50% of patients had undergone DRE during a general medical exam. Most importantly, more than half of the examiners were not confident enough in performing the exam. The study states that guidelines should be established for the standardization of performance of DRE which should be incorporated in medical school and clinician teaching programs.

It is reasonable to assume that lack of training regarding anorectal disorders in medical school would lead to the expected low diagnostic accuracy rates in specialties other than general surgery.

In addition, as most operations for benign anorectal disorders have become outpatient procedures, examination of a patient with such conditions on a “ward rotation” has become a rare occurrence. This is possibly where the educational importance of the outpatient colorectal clinic becomes significant for medical students.

Attending the outpatient colorectal clinic under direct supervision and mentoring of a colorectal specialist theoretically exposes the trainee (student, surgical resident) to a fair number of benign anorectal conditions. The trainee is taught how to obtain an anorectal history and perform an examination in a standardized fashion. A comprehensive assessment of the condition, a diagnosis is made and a treatment plan is proposed and discussed with the patient. In many cases, operative and non-operative treatment options exist; benefits and complications of each option are explained and a decision for treatment is made.

Our study demonstrated that medical students attending the outpatient colorectal clinic had better overall diagnostic accuracy for benign anorectal conditions than those that did not. In addition, higher rates of accuracy were demonstrated for common conditions such as prolapsed internal hemorrhoids, thrombosed external hemorrhoids, fissure and anal tags. Accuracy for full-thickness rectal prolapse was similar and relatively high; this may be explained by the fact that this disorder may provide an impressive visual stimulus, is explicit and highly diagnostic. Grucela et al. demonstrated high diagnostic accuracy for rectal prolapse in most non-surgical specialties [1].

Our study is limited by a small number of students accrued and by the fact that diagnoses were made by viewing images only. Diagnoses made after live patient encounters as well as adding a digital rectal examination component in the study would be the next logical step in studying diagnostic accuracy. Furthermore, we did not assess the ability to retain levels of diagnostic accuracy over time. Miller et al. studied diagnostic accuracy in surgical trainees at different levels of training, and found a trend of diminished diagnostic accuracy over time [2]. Therefore it is not known whether our students would be able to make accurate diagnoses of anorectal conditions after graduation and specialization. This remains to be studied. Finally, since there was only one clinical tutor, who also performed the lecture on anorectal disorders, there is a potential for teaching outcome bias in this study.

How does one become proficient in the diagnosis of benign anorectal conditions?

The mechanism of learning may be intriguing itself. Benign anorectal disorders have visual clues; the prolapsed columnar epithelium in internal hemorrhoidopathy; the glistening of the squamous epithelium in an external thrombosed hemorrhoid, the concentric folds of a full-thickness rectal prolapse and sentinel tags of fissures are among a few. Despite the critical value of many of these visual features in the diagnostic process, little is known about the manner in which physicians integrate this information into their diagnostic formulations. Verbal information during history taking when evaluating patients may also contribute to the making of the diagnosis [9]. Digital rectal examination is an important part in the making of the diagnosis of anorectal disorders as well, despite the plethora of diagnostic modalities available, such as endoscopy, manometry, ultrasound and advanced imaging. There is good correlation between DRE and other diagnostic modalities, such as anorectal manometry [10].

Finally, it has been demonstrated that the addition of full-time colorectal faculty in a surgical department may lead to a significant increase in exposure of surgical trainees to anorectal procedures [11]. One may extrapolate and submit that incorporating attendance of an outpatient colorectal clinic during a surgical clerkship, under supervision of a full-time colorectal specialist, may provide students with the opportunity to familiarize themselves with highly prevalent conditions such as benign anorectal disorders.

## Conclusion

Attendance of an outpatient colorectal by medical students was associated with higher diagnostic accuracy of the majority of common benign anorectal conditions. As most of these conditions are frequently seen by non-colorectal specialist physicians, we believe it would be of benefit to medical students who will subsequently train in these specialties to receive education regarding these conditions at the undergraduate level. Hopefully, this would lead to better care of their patients as a result of a prompt and accurate diagnosis.

## Authors’ information

Constantine Spanos, Assistant Professor of Surgery, Aristotelian University.

Apostolos Tsapas, Associate Professor of Medicine, Aristotelian University.

Manolis Abatzis-Papadopoulos, Graduate of the School of Medicine, Aristotelian University.

Eleni Theodorakou, Graduate of the School of Medicine, Aristotelian University.

Giorgios Marakis, Professor of Surgery, Aristotelian University.
